# Bibliographical Notices

**Published:** 1851-01

**Authors:** 


### BIBLIOGRAPHICAL NOTICES.

*Transactions of the American Medical Association, instituted* 1847. Vol. III. Philadelphia, 1850. pp. 499.

(Continued.)

REPORT ON MEDICAL EDUCATION.

This Report which treats of a subject, which has been regarded by the Association as one of primary importance, in its connection with the existing and future state of medical science in America, covers but seven pages of the Transactions. The copiousness of former reports is considered by the Author as a sufficient reason for omitting a reference to many of the more special subjects which have claimed the attention of previous committees. But, notwithstanding the labors of his predecessors are regarded as so full, as to leave little to be accomplished, the Author of the Report appears to consider that they have left the subject in a mist of obscurity.

"The records of the discussion thus far embrace, as in most cases where practical subjects have been submitted to abstract investigation, a somewhat intricate intermingling of *facts* and *opinions*.

"The *facts* include a statistical history of the schools, professors, students, and graduates; their situation, quantity, kind and means of instruction; preliminary and ultimate requirements, modes of examination, and other important particulars.

"The *opinions* relate chiefly to conclusions thought to be deducible from the *facts*. So far as they have assumed a definite form, they assert, first in general terms, that the system of medical education in this country is defective. Second, that it is so because the number of schools is too great, their situation (in many cases) bad, their instructors too few, their students and graduates too many, the time devoted to instruction too short, the quantity too limited, the quality too superficial, the bestowal of honours too profuse, and the mode of bestowal too unrestricted; all this involving, inevitably, a depreciation of the profession, and an impairment of the dignity, honour, and usefulness of the healing art."

How far these facts and opinions accord with the Reporter's own views, we are not informed, nor has he attempted to unravel the "somewhat intricate intermingling of facts and opinions" which he has discovered in former reports. To our minds he has, however, deduced sufficient material, from the labors of his predecessors to have formed a text for copious and profitable discussion. How stand the statistics of the schools for the past year? how many new ones have sprung into existence? where are they located, and what their means of giving a thorough medical education? Have the schools made any advance within the year in the means of instruction? Are practical anatomy, practical pharmacy, and clinical teaching, more regarded than in former years? Have the preliminary and ultimate requirements, and the mode of examination been improved or modified in any of the schools? Have any of them increased their number of Professors, lengthened their sessions, or improved the instruction either in quantity or quality? Is the bestowal of their honors more guarded and limited, or have they, or any of them, modified their courses in any essential particulars, and thereby added to "the integrity, honor, and usefulness of the healing art?"

From the Author's admitted appreciation of the labors of his predecessors, one would think that all these questions would have naturally arisen, in following up what they had commenced. Certainly the Association had a right to expect that such information would have been laid before them, by a committee to whom they had entrusted the duty of setting forth the progress which medical education was making within its limits. The Association had issued its recommendations, based upon glaring and increasing deficiencies in the courses of the medical schools, as officially reported to them, it was their right to know how far these recommendations had been heeded, and by what bodies they had been disregarded.

Such schools for instance, as had extended their terms of instruction, or shown a disposition to improve upon former plans, should have been made known, and those which had declined to follow out the requisitions of the Association, should have been represented in the Report, in their own defence.

Instead of this we have a general acknowledgment of the good will of the schools, which is thus complacently set forth.

"The schools have been disposed to meet the efforts of the Association in a spirit of cordial co-operation. Some of them have attempted to carry into practice its most important recommendations; and almost all have manifested a desire to contribute to the improvement of professional education. In no instance has any one of them, so far as the Committee can learn, assumed a hostile aspect towards the Association or its objects. Whatever reproaches may have been uttered against them for deficiency or delinquency, they have reason to feel proud that their relation to the Association has been, from the first, one of harmony and good will; that they have identified themselves with it, not to thwart or defeat its great designs, but to further their fulfilment; not merely for their own honor and their own interest, but for the advancement of science and the good of their fellow men."

Now this language may lull the indifferent or listless spectator of passing events, into the fond belief, that great things are being done for medical education. Some schools have, we are told, *attempted* to carry into practice the most important recommendations of the Association; and almost all have *manifested a desire* to contribute to the improvement of professional education. Not one has assumed a *hostile aspect* towards the Association or its objects." This is certainly very well, as far as it goes. It is something for the representatives of the great medical community of the Union, to be thought well of by the schools, and not to be openly opposed by any single one of them; but it is far more important that their fruits should manifest a "cordial cooperation" with the objects which the body has at heart, in elevating the standard of medical education, and thereby increasing the usefulness and respectability of the profession.

Upon the several recommendations of the Association, in regard to medical education, the report is not altogether silent. In regard to preliminary education, great deficiency is acknowledged, which "should be equally shared by the profession, the schools, and the students."

The report further remarks:

"The Association has attempted to obviate this difficulty by urging upon the profession and the schools, certain recommendations, which as yet have not been fully complied with. Neither of the parties most interested seems willing to assume the responsibility. The student dislikes to have his self-complacency offended; the practitioner fears to hazard the good will of the student; and the school, it is feared, is often too anxious, lest the portal of some active rival should be found of easier access than its own. Hence, with the defect generally admitted and deplored, it does not appear that hitherto much has been done towards applying any efficient remedy."

On the subject of lengthening the lecture terms of the schools the Report holds the following language.

"The charge has been somewhat pertinaciously repeated that the student, after entering the schools, is often imperfectly and insufficiently taught; that there are schools badly situated, scantily officered, and meagrely equipped—unable, in short, to fulfil their promise to provide a proper course of public instruction. The Committee are not in possession of the name of any particular school against which, justly or unjustly, this charge has been openly and directly alleged. If there be a single school fairly deserving such an imputation, it is hoped no false compassion will interpose to shield it. After three years of consultation and discussion, it surely cannot be deemed unreasonable that this Association should discriminate between those schools which have its confidence and those which have not.

"Practically, the Association has advised an increase in the number of instructors, and an extension of the period of instruction. It is not known that either of these recommendations has been generally adopted; as to the last, it would seem that the original design of the Association, which, as the Committee apprehend, contemplated a lengthening of the term of lectures without materially increasing their number (on the ground that too many were crowded into too short a time), has been somewhat misunderstood. Since, of late, it has been argued that the *completeness* of a course of lectures necessarily depends upon its duration, and that this is an indisputable advantage of the six over the four months' course. The schools also appear to differ in their understanding of this point; some of them having increased both the term and amount of instruction, while others have extended the former alone. Thus, in the institution selected by the committee of 1849, 'as a model of the schools of our own country, whose rules and requirements are believed to be quite as full and extensive as those of any other institution, and whose trustees and professors seem to be as well disposed as any others to accede to the general wish of the profession, as expressed through this Association, to favour proper measures of reform'—although the lecture term is one of six months, the aggregate amount of instruction, as reported, is really less than in another school whose course continues but four and a half months. The Committee would suggest, therefore, that it should be fairly stated, by those schools in which the session is prolonged, whether there is, or is not, a proportional increase of the amount of instruction."

Is the Professor who penned this section really ignorant of the facts, which his predecessors have so clearly set forth, in regard to the *general* deficiency of medical schools, to furnish that full and complete course of instruction, which the requirements of the age, and the advancing spirit of medical science demand? Does he not know that the competition amongst the schools has sadly lowered the standard of education, and that fears are entertained that one school admits and passes unqualified persons, "lest, to use his own language, the portal of some active rival should befound of easier access than its own?"

Why cite particular schools in which defective courses are given; in which no adequate clinical instruction is furnished; in which practical anatomy is lightly passed over, and in which a diploma is granted without that full and complete study which is required by the public voice of the whole profession; when the facts and statistics are spread out before the public, so that he "who runs may read"? The duties of the Association are not to single out particular institutions, and to hold them up to invidious distinction; but, to sustain and cherish those which, acknowledging their defects, manifest a determination to improve their means of instruction, and to co-operate with it in the noble work of elevating the standard of education, irrespective of narrow and selfish interests. Instead therefore of pleading for existing abuses, and endeavoring to make it appear that one school can give more instruction in four months and a half, than another can in six months, we would suggest that the efforts of future committees should be directed to sustain the previous action of the Association, and to advance the good cause of progress in which it has embarked.

That there has been just ground for the complaints which have been made by previous committees on this subject, we are fully convinced; and we cannot but regret that after three elaborate and high-toned reports upon it, in which a more elevated ground has been assumed, calculated to incite our medical teachers to high and noble effort in the responsible position which they have assumed, that the paper before us should fall below its predecessors, and should endeavor to palliate those defects and evils which must be candidly exposed and acknowledged before a remedy can be applied.

To show the effect produced upon the minds of our transatlantic brethren, by the revelations of former committees upon this subject, it may not be inappropriate to introduce here a brief notice of the reports upon Medical Education, introduced into a review of our transactions, which appears in the British and Foreign Medico-Chirurgical Review, for Oct., 1850, with the remark, that although something is to be allowed for the ignorance of the profession abroad, in regard to the actual state of medical knowledge in this country, yet there is, in the main, much reason for the strictures contained in the following extract:

*Reports of the Committees on Medical Education.*—This is properly deemed, by a body of enlightened men, having the elevation of the *status* and increase of the utility of their profession at heart, as the most vitally important subject that can engage attention. We were not prepared for some of the disclosures here made, and certainly feel somewhat more reconciled to the delays which have taken place in the reform of our own institutions, after contemplating the infinitely worse condition of those of our neighbours. We will summarily indicate the chief defects:—1. The Student commencing attendance on lectures is not required to give any proof of preliminary education. 2. The courses of lectures are only of three or four months duration for two years, the various subjects, too, being treated of, not successively, but simultaneously; 3. Medical colleges obtain charters with great ease in the different States, and there are already thirty-eight such institutions, invested with the double power, of teaching and conferring the degree of M.D. A fierce competition prevails among these bodies, and various inducements are held out to students by some of them, such as low prices and long credit, in order to swell the number of their pupils. 4. Not only is the duration of the session ludicrously short, but several subjects are ill-taught, and others not taught at all. Thus, practical chemistry and pharmacy meet with little favor; while in few of the institutions are lectures delivered on botany, or on comparative or pathological anatomy; and practical anatomy itself is so neglected, that some of the colleges announce in their circulars, attention to it will not be made obligatory! The number of professors engaged in teaching in the European schools is more than double that employed in the American ones, and sometimes four or five times as great. 5. Hospital attendance seldom forms any portion of the system of instruction, and the reporters say, that even in large cities like New York and Philadelphia, not more than one in ten of the pupils attend regularly at the hospital. Moreover, in most of the colleges all attempt at such teaching is eschewed, and a wretched substitute called "College Clinics" resorted to, at which, perhaps, the pupil may see the same patient but once or twice, when he comes for advice. 6. The system of examination is bad; for the professors examine their own pupils, and decide upon their admissibility. In some of the institutions these examinations are quite private, each professor testing the capabilities of the candidate alone and apart from his colleagues. "Three out of seven, or two out of six, negative votes are usually considered necessary for rejection. Thus a candidate may be wholly unprepared in one or two of the most important branches—anatomy and surgery for instance—and yet receive his degree!" Lastly, if a pupil finds himself unable to undergo even such an ordeal as this, he need not despair, but may at once set himself up as doctor, only two or three of the States imposing any examination, or even any education at all, as a necessary preliminary to practice.

Mortifying as it may be to our national pride, to see American schools thus held up to reproach in the pages of one of the first medical periodicals of the times, it is impossible to escape from the position in which our own acknowledged defects have placed us; and, unless we expect tamely to fall back in the race for scientific distinction in which other nations are engaged, we must raise the tone of our professional education and literature. Instead of apologizing for existing evils as incident to our political system, and to the habits and genius of our people, we must join hands in strenuous and persevering efforts, to advance American medicine to that high point of excellence and utility of which it is as susceptible here, as in any other portion of the world.

That the American Medical Association may not relax its efforts in this good work, is the strong desire of the friends of medical progress both here and abroad. And that it is not yet prepared to do so, was, we think, fully verified by the general feeling of disappointment which pervaded the Association, on the reading of the report before us, as well as by the fact that it appears before the profession with the name of the chairman of the committee alone; two of the members, who were present at Cincinnati, Drs. Blatchford and Roberts, publicly dissenting from its conclusions, and declining to append their names to it.

(To be continued.)

---

*Woman; her Diseases and Remedies. A series of Letters to his Class.* By CHARLES D. MEIGS, M. D., Professor of Midwifery, &c., in the Jefferson Medical College at Philadelphia, &c., &c. *Second edition, revised and enlarged.* Philadelphia, Lea & Blanchard, 1851.

The first edition of Dr. Meigs' work on Woman and her Diseases, was noticed at some length in the Examiner for January,

1848. Of this second edition, "revised and enlarged," we have here only to repeat the favorable opinion previously expressed of its general bearing and value, referring our readers to the more detailed comments upon some of the author's peculiarities of style and doctrine which were then indulged in. The early demand for another edition attests the popularity of the book, and seems to leave no longer doubtful Dr. Meigs' anticipation, that the familiar and vivacious style of treating medical subjects, which he has adopted—however critics may carp—is likely to "prove agreeable to the brethren, so as to meet their approbation."

---

*A Chart of the Signs furnished by Auscultation and Percussion, and of their Application to the Diagnosis of Diseases of the Lungs.*

*A Chart of the Signs furnished by Auscultation and Percussion, and of their Application to the Diagnosis of Diseases of the Heart. Revised and arranged by* P. CLAIBORNE GOOCH, A. M. M. D., etc. *From the French of* O. BELLINGHAM, M. D. P. Richmond, Va. Morris & Bro.

In these two charts we have a bird's eye view of the physical signs and their causes, as they occur in the physiological and pathological conditions of the thoracic viscera, together with observations upon them.

In casting our eyes over them we have been pleased with the plan of arrangement, and only wonder that it has not occurred to others before to construct such an exposition of these subjects for office reference. There are a few points, however, in which we differ, and think the American editor might have improved upon the original; for instance: In explaining the cause of the crepitant râle in pneumonia, the author describes it as produced "by the passage of air, in inspiration, through a muco-sanguinolent liquid secreted in the air cells and minute bronchial tubes." We know that for this there is high authority, but it has nevertheless, always seemed to us that the explanation of Dr. Carr, of New York, was more satisfactory. This observer ascribes it to the separation of the inner surfaces of the air vesicles, which had become adherent to each other during expiration, in consequence of the viscid, but scarcely *fluid* secretion, with which their interior is lined. This view seems confirmed by the observations of Messrs. Rainey and Hutchinson, by whom it is shown that each bronchial tube terminates in a cluster of cells, instead of a single vesicle, as described by Reiseissen. This explanation of the cause, however, does not detract from the value of the sign as a means of diagnosis.

There are one or two points of expression which we think might also be improved by substituting others; for instance, metallic *tinkling*, for metallic *tingling ;* the word *nipple*, instead of teat, &c., &c. These will doubtless meet the eye of the translator in preparing a new edition. The text of the author is very fairly rendered.

The Charts, it should be mentioned, are made up from the works of Laennec, Williams, Barth & Roger, Gerhard, Spittal, and other high authorities, and we have no question but that they will prove very acceptable, as well as useful to the profession.

---

*The Family and Ship Medicine Chest Companion: being a compendium of Domestic Medicine, Surgery, and Materia Medica, with Directions for the Diet and Management of the Sick Room, &c., &c. Selected from standard works by a* PRACTISING PHYSICIAN. Philadelphia, Lindsay & Blakiston, 1851.

We have so often expressed our opinion of the tendency of the unrestricted circulation of *household* works on medicine, that it is unnecessary here to reiterate it. Under many circumstances, however, where medical aid is beyond reach, such books are undoubtedly desirable and useful, and we have great pleasure in recommending this little manual as an excellent work of the kind. The publishers very properly represent "the object of the work to be, to afford assistance to those who are removed from medical or surgical aid, but *in no way* to delegate the treatment and care of the sick to the inexperienced, when other assistance can be obtained."

